# Prefrontal Hemodynamic Functions during a Verbal Fluency Task in Blepharospasm Using Multi-Channel NIRS

**DOI:** 10.1371/journal.pone.0150804

**Published:** 2016-03-04

**Authors:** Chen-Yu Shen, Yong-Jun Wang, Xiao-Qian Zhang, Xiao-Min Liu, Xia-Jin Ren, Xiang-Yun Ma, Jing-Jing Sun, Kun Feng, Gao-Xiang Sun, Bo Xu, Po-Zi Liu

**Affiliations:** 1 Medical Center, Tsinghua University, Beijing, China; 2 YuQuan Hospital, Tsinghua University, Beijing, China; 3 Tianjin Anding Hospital, Tianjin, China; Hangzhou Normal University, CHINA

## Abstract

Blepharospasm (BSP) has a morbidity of 16 to 133 per million and is characterized by orbicularis oculi spasms. BSP can severely impact daily life. However, to date, its pathophysiology has not been clearly demonstrated. Near-infrared spectroscopy (NIRS) is a portable, non-invasive, and high time resolution apparatus used to measure cerebral blood flow. This study aimed to investigate the hemodynamic response patterns of BSP patients and determine whether BSP alone can be an attributional factor to influence the function of the prefrontal area using a verbal fluency task (VFT) and NIRS. Twenty-three BSP patients (10 males and 13 females) and 13 healthy controls (HC; five males and eight females) matched by gender and education were examined using NIRS. BSP patients were divided into two groups based on the presence or absence of depression and anxiety symptoms. A covariance analysis was conducted to analyze differences between the three groups and reduce the influence of different ages and educational levels. Bonferroni was used to process the post hoc test. The bilateral orbitofrontal area (ch36, 39, and 41; *P<0*.*01*) exhibited a lower activation in BSP patients without psychiatric symptoms compared with HC. This study is the first report to identify the prefrontal function in BSP using NIRS. Our findings indicate that BSP alone may cause a hypoactive hemodynamic performance in the prefrontal cortex in the absence of psychiatric symptoms. These findings provide evidence to support novel pathophysiological mechanisms of BSP.

## Introduction

Blepharospasm (BSP) is a common adult-onset focal dystonia, which is characterized by bilateral and typically symmetrical and synchronous orbicularis oculi spasms[1,2]. The prevalence of BSP ranges from 16 to 133 per million[3]. However, the prevalence of BSP in China remains unclear. Wind and/or light may exacerbate clinical symptoms, whereas attentional shifting and repose comprise alleviating factors. BSP patients have a higher frequency of eye diseases, such as blepharitis and kerato conjunctivitis[4], and dystonia symptoms often spread to the orem and ibular region (Meige’s syndrome)[5]. Although BSP may have a serious impact on the quality of life, the pathophysiology of this disease has not been clearly identified to date.

Several studies have identified an increased prevalence and/or severity of psychiatric conditions in patients with BSP, such as depressive disorders[6], generalized anxiety disorder[7], and obsessive-compulsive disorder[8,9]. Psychiatric comorbidity in BSP patients was originally ascribed to the experience of embarrassment and disfigurement[10]. Lencer and colleagues suggested that psychiatric symptoms often precede movement disorders, even by several years[11]. Recent studies have demonstrated that striato-thalamo-cortical circuit dysfunction may play an important role not only in dystonia but also in affective disorders[12,13].

With the development of neuroimaging techniques, several studies on BSP patients using functional magnetic resonance imaging (fMRI) have demonstrated aberrant activation patterns in the bilateral frontal and orbitofrontal gyri, which were correlated with cognitive and mood symptoms[14]. In addition, BSP studies using positron emission tomography (PET) have demonstrated consistent abnormal patterns in several cortical and subcortical areas, including the inferior frontal lobe[15]. These previous studies have all reported prefrontal cortex dysfunctions. Thus, it is reasonable to assume that BSP patients have abnormal prefrontal hemodynamic responses.

Importantly, previous studies have rarely controlled for psychiatric symptomatology. Thus, we cannot conclude whether prefrontal cortex dysfunction is a result of BSP alone. Moreover, none of the previous studies systematically combined functional neuroimaging with cognitive tasks, and only the resting state[16] or simple movement tasks, such as whistling, were employed[17]. Finally, to the best of our knowledge, there is a lack of near-infrared spectroscopy (NIRS) studies regarding this disorder, despite its portability, non-invasiveness, low-cost, and high time resolution compared with fMRI and PET.

To address this issue, the present study aimed to investigate the hemodynamic response patterns of BSP patients and determine whether BSP alone can be an attributional factor to influence the function of the prefrontal area using a verbal fluency task (VFT) and NIRS. We hypothesized that similar to individuals with psychiatric disorders, BSP patients would exhibit aberrant prefrontal hemodynamic responses that could be identified using NIRS. This finding could facilitate an understanding of the neurobiological or psychobiological mechanisms of BSP.

## Materials and Methods

### Subjects

Twenty-three primary BSP outpatients excluding segmental dystonia (10 males and 13 females) were recruited from the Psychiatry Department of Yuquan Hospital. Thirteen healthy controls (five males and eight females) without a medical history of neurological or psychiatric disorders were also recruited via advertisements. BSP patients were divided into two groups: G1 (*n* = 13, seven males and six females) and G2 (*n* = 10, six males and four females), depending on the presence or absence, respectively, of depression or anxiety symptoms. All patients in the G1 group met the diagnostic criteria for major depressive disorder and general anxiety disorder based on the Diagnostic and Statistical Manual of Mental Disorders fourth edition (DSM-IV).

Patients with other psychiatric symptoms or disorders, chronic substance abuse, or a severe medical illness related to cognitive function were excluded. All subjects were matched for gender and educational level, while patients in G2 group and HC were matched for Hamilton Depression Rating Scale (HAMD, 24-item, Hamilton, 1960) and Hamilton Anxiety Rating Scale (HAMA, Hamilton, 1956) scores. This study was approved by the Ethics Committee of Yuquan Hospital, and written informed consent was obtained from all participants.

### Activation task

The activation task comprised of a semantic category version of the verbal fluency task (VFT). Relative concentrations of oxy-hemoglobin (oxy-Hb), deoxy-Hb, and total-Hb were measured during the VFT, which consisted of a 30-second pre-task baseline, a 30-second VFT, and a 30-second post-task baseline. The subjects were instructed to verbally list as many items as possible that belonged to a specific semantic category (vegetables, family applications, such as computers, four-foot animals, and fruits). The number of correct words generated during the task period was recorded as the evaluation of cognitive performance.

### Assessment

All subjects were evaluated by an experienced psychiatrist according to the Structured Clinical Interview for DSM Disorders (SCID). HAMD and HAMA were completed to determine the severity of depression and anxiety. Demographic information was collected using questionnaires developed by our group.

### NIRS measurement

**Fig 1:**
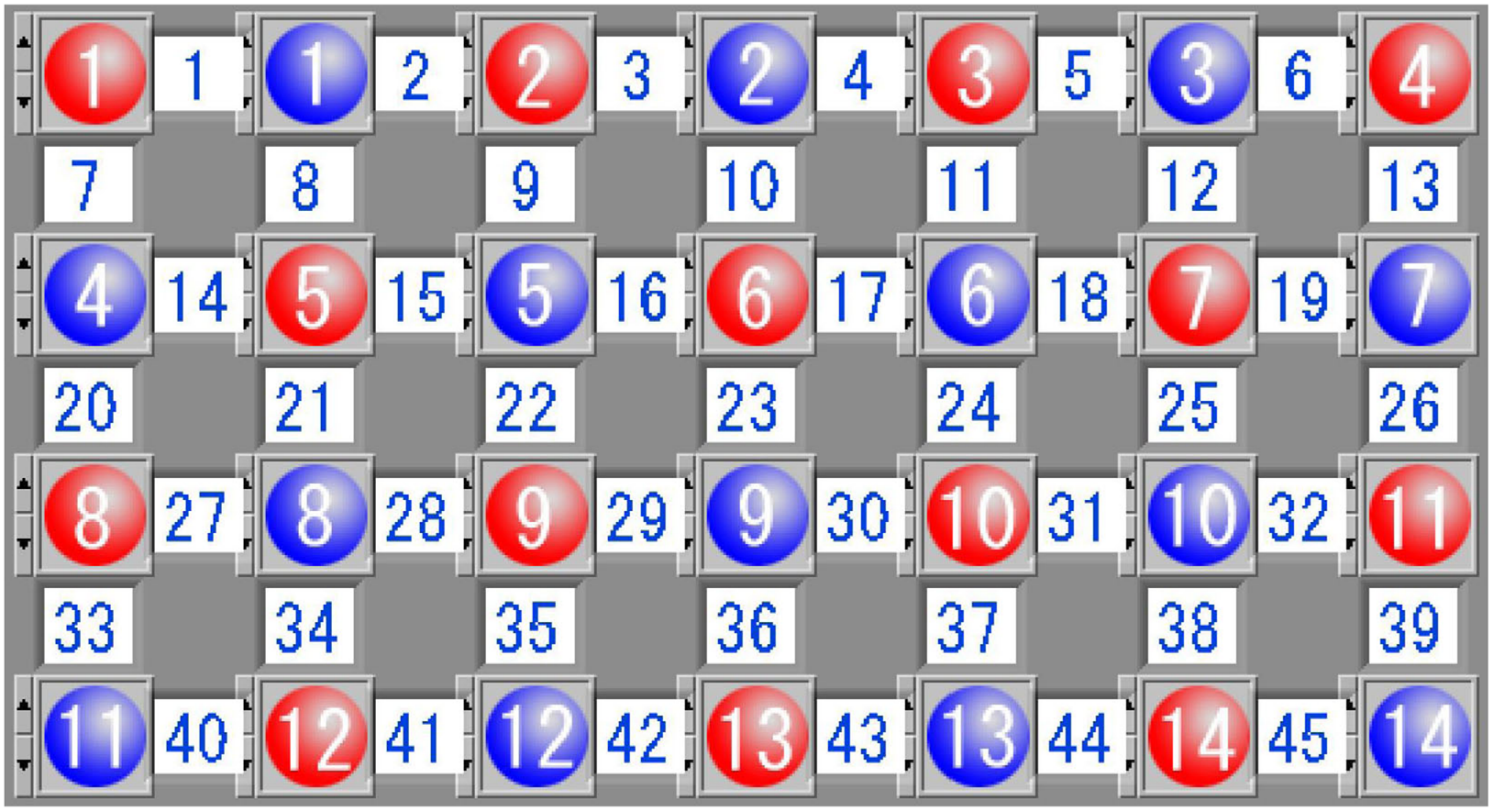
Fourteen pairs of probes comprised 45 channels. The red and blue numbers represent emission and detector probes, respectively. The bilateral DLPFC and the orbitoprefrontal cortex are covered by these channels.

A 45-channel near-infrared spectroscopy system (FOIRE-3000, Shimadzu) was applied to measure hemodynamic responses during the VFT task. Three different relative concentrations (oxy-Hb, deoxy-Hb, and total-Hb) were obtained by the FOIRE-3000 based on the modified Beer-Lambert Law. Fourteen emission probes and 14 detector probes were placed on the forehead of each participant (Fig 1), and each pair of emission and detector probe consisted of a channel. The bilateral DLPFC and the middle prefrontal cortex were covered by these channels. The lowest probes were positioned along the Fp1-Fp2 line according to the International 10–20 system of electroencephalogram electrode placement[16].

### Statistical analysis

One-way analysis of variance (ANOVA) was used to analyze demographic and clinical variables, as well as the VFT performance among the three groups, with the exception of the gender item (contingency table Chi-square analysis). The oxy-Hb increase was calculated by the difference between the mean oxy-Hb of the task and the pre-task period. To reduce the influence of age and education level, a covariance analysis was used to analyze the differences among the three groups. Bonferroni was used to process the post hoc test. Homogeneity of variance was tested to compare the distribution shape of the different groups. Oxy-Hb was selected as the analysis index because of its reliability to exhibit changes in regional cerebral blood flow[18,19]. The Statistical Package for Social Sciences (SPSS, IBM Corporation, Armonk, NY, US) version 21.0 in windows was used for analyses.

## Results

### Demographic and clinical data of patients and controls

The demographic characteristics of the three groups are presented in Table 1. There were no significant group differences in gender, disease duration, education level, or motor symptom severity, as assessed by the Jankovic Rating Scale (JRS). Furthermore, the HAMD and HAMA scores between patients in the G2 group and HC were not significantly different. Age between patients in the G1 group and HC, as well as age between patients in the G2 group and HC, were significantly different (Columns B to G in S1 Dataset).

**Table 1:** Demographic and clinical data of BSP patients and healthy controls.

Demographics	BSP with D/A (G1)	BSP without D/A (G2)	HC	ANOVA Sig.	G1 *vs*. G2 Sig.	G1 *vs*. HC Sig.	G2 *vs*. HC Sig.
Age (years)	51.15 ± 6.99	48.50 ± 11.25	35.85 ± 11.08	0.001	0.541	<0.001	0.004
Gender (female/male)	6/7	4/6	5/8	0.917	-	-	-
BSP duration (years)	2.88 ± 1.70	3.10 ± 1.45	-	0.751	-	-	-
Education level (years)	12.92 ± 3.01	13.60 ± 3.53	15.85 ± 2.94	0.061	-	-	-
JRS	5.15±1.57	4.90±1.52	-	0.701	-	-	-
HAMD	14.38 ± 4.88	5.80 ± 2.35	6.46 ± 1.66	<0.01	<0.001	<0.001	0.641
HAMA	15.54 ± 3.93	6.90 ± 2.56	5.38 ± 2.02	<0.01	<0.001	<0.001	0.235

BSP: blepharospasm, D/A: depression and anxiety symptoms, HC: healthy controls, JRS: Jankovic Rating Scale, HAMD: Hamilton Depression Scale, HAMA: Hamilton Anxiety Scale

### Task performance

The number of words generated during the VFT task by the three groups is presented in Table 2. Compared with the HC, the BSP patients (in both the G1 and G2 groups) generated a significantly different mean number of words during the VFT task; an ANOVA indicated that three of the categories were significantly different with the exception of vegetables. Least significant difference (LSD) was used to compare two of the three groups, and differences between patients in the G1 group and HC were identified in all three categories (*P*<0.01, *P* = 0.013, and *P* = 0.002). The G2 group and HC were only significantly different regarding family applications (*P* = 0.003). None of the four categories were significantly different between the G1 and G2 groups (*P*>0.05) (Columns H to K in S1 Dataset).

**Table 2:** Number of words generated during the VFT task by BSP patients and healthy controls.

	BSP with D/A (G1)	BSP without D/A (G2)	HC	ANOVA Sig.	G1 *vs*. G2 Sig.	G1 *vs*. HC Sig.	G2 *vs*. HC Sig.
Vegetables	8.77 ± 2.49	10.7 ± 3.30	10.92 ± 3.28	0.160	-	-	-
Family applications	7.23 ± 2.46	7.90 ± 2.23	11.31 ± 2.81	0.001	0.534	<0.001	0.003
Four-foot animals	7.85 ± 2.12	8.50 ± 2.84	10.46 ± 2.67	0.035	0.543	0.013	0.074
Fruits	8.23 ± 2.46	9.50 ± 2.76	11.77 ± 2.95	0.008	0.276	0.002	0.056

BSP: blepharospasm, D/A: depression and anxiety symptoms, HC: healthy controls

### Group comparisons of NIRS data

#### G1 *vs*. G2 *vs*. HC

Changes in the oxy-Hb concentration were homogeneous in 38 channels (*P*>0.05), with the exclusion of ch 3, 6, 13, 17, 19, 20, and 43. Significant interactions between age and group were identified in two channels (ch 9 and 14, *P*<0.05). None of the 38 channels exhibited significant interactions between educational levels and groups. Three of the left channels (ch 36, 39, and 41) were significantly different among groups (*P*<0.05), whereas ch 22 exhibited a significant difference among ages (*P*<0.05). None of these channels exhibited a significant difference among educational levels (*P*<0.05).

#### Post hoc test (Bonferroni)

**Fig 2:**
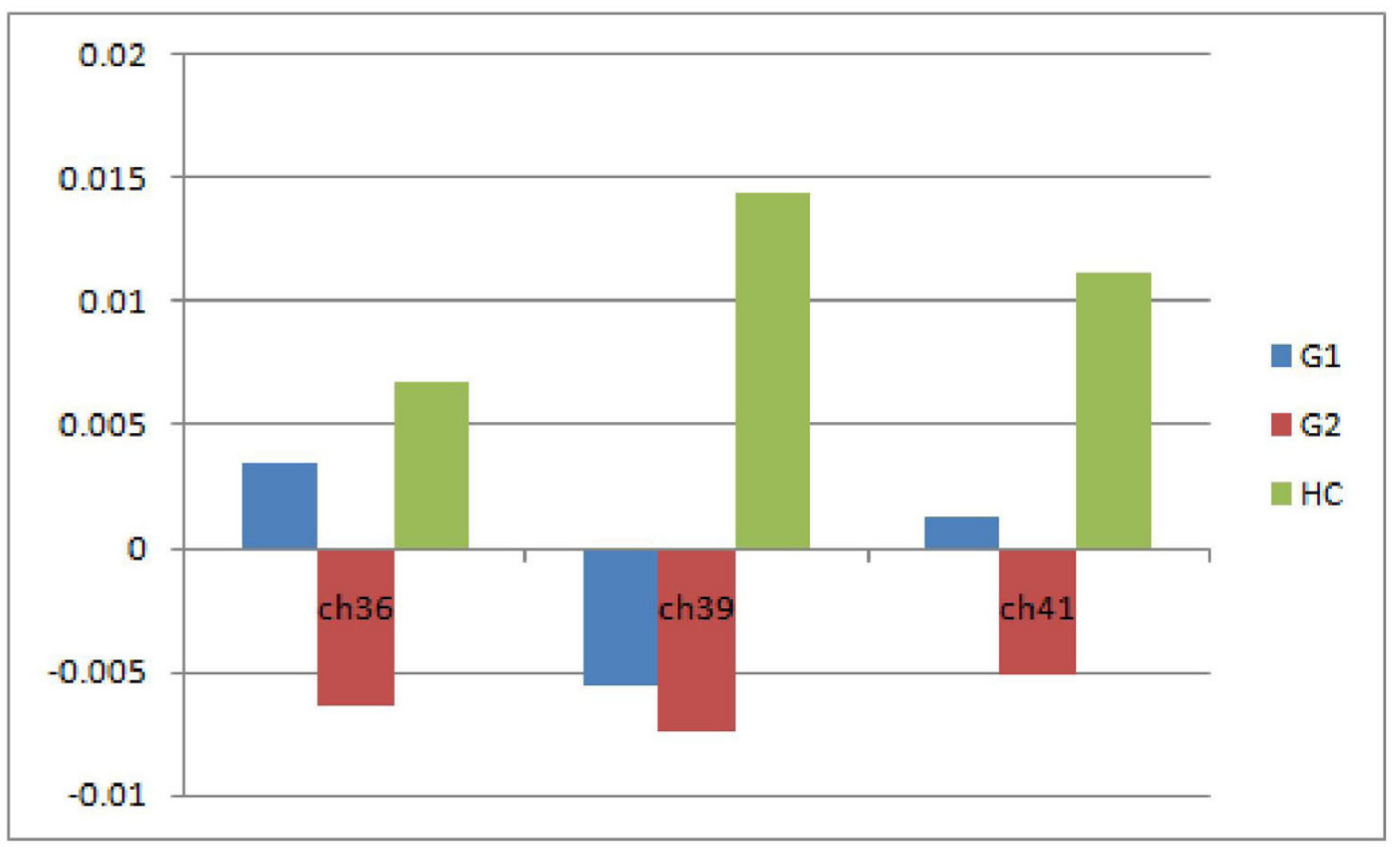
Histogram of the average oxy-Hb concentrations of the three channels. Both the G1 and G2 groups exhibited smaller oxy-Hb increases compared with the HC group.

Ch 39 was not significantly different between the G1 and G2 groups (*P* = 1.000). However, both groups were significantly different from HC (*P* = 0.038, 0.018). Ch 36 and 41 were significantly different between the G2 and HC groups (*P* = 0.013, 0.035). All patients in the G2 group exhibited significantly smaller oxy-Hb increases compared with HC in the three channels during the VFT task. No significant difference was identified between G1 and HC in Ch 36 or 41 (*P* = 0.849 and *P* = 0.251); however, the average oxy-Hb concentration of the G1 group was lower than the HC group. Both ch 36 and 41 were not significantly different between the G1 and G2 groups (*P* = 0.094, 0.984; Fig 2) (Columns L to BD in S1 Dataset).

## Discussion

To identify the pathomechanism of BSP, this study explored the hemodynamic responses of the prefrontal cortex using NIRS during the semantic category VFT in BSP patients without depression and anxiety symptoms. These patients were compared with HC and patients that exhibited these emotional symptoms in the BSP group. To the best of our knowledge, this study is the first report that used NIRS combined with a cognitive task in BSP patients. It is also the first investigation to control for psychiatric symptoms in BSP patients examined via neuroimaging techniques.

### Impaired cognitive function in BSP

The VFT is a standard procedure used to investigate cognitive and linguistic abilities[20]. It has been proven to be a valuable tool to examine executive control processes in psychiatric disorders related to cognitive impairment[21,22].

BSP patients with depression and anxiety symptoms performed significantly worse than HC in the last three categories. Although BSP subjects without depression and anxiety symptoms were only significantly different regarding family applications, the average number of words was lower than HC subjects. These findings suggest that the disrupting effects of BSP alone could cause cognitive and executive dysfunction. The ability to retrieve serials of nouns based on a common criterion is primarily related to frontal lobe function[23]. The poor performance of BSP patients indicates dysfunction of this area. There was no significant difference between BSP subgroups. This finding may demonstrate that psychiatric problems have a minimal impact on cognitive performance, especially the working memory domain.

To our knowledge, studies have rarely included cognitive evaluations for BSP patients, especially with the VFT. Furthermore, previous results have been inconsistent. Vidailhet *et al*. reported their findings for a group of 22 primary dystonia individuals, in which they did not identify abnormalities in the Mini-Mental State Examination scores[24]. In contrast, Aleman *et al*. assessed the cognitive function of 20 BSP patients matched with 17 controls for age, educational level, and affective symptom severity. The patient group exhibited a significantly worse performance in a series of cognitive performance tasks, which reflect different cognitive aspects[25]. Based on Romano’s research, patients with BSP or cervical dystonia (CD) may have impairments in specific cognitive domains, including working memory, which reflect cortical and subcortical changes[26]. Similarly, Duane and Vermilion also identified evidence of executive and visual memory deficits in primary dystonia[27]. These findings support our research.

### Hypoactivation of the bilateral orbitofrontal cortex in BSP without depression and anxiety symptoms

The present study indicates that prefrontal oxy-Hb activation during the VFT was significantly lower in BSP patients without depression and anxiety symptoms compared with matched HC. The most significant (*P*<0.01) areas of the prefrontal cortex comprise the bilateral orbitofrontal cortex (ch 36, 39, and 41; Fig 1). This finding may suggest that despite the absence of psychiatric symptoms, BSP alone could cause hypoactivation. The reduction in oxy-Hb activation during the cognitive task period suggests that the prefrontal cortex of BSP patients was not able to obtain an adequate increase in blood supply to compensate for the consumed oxygen, which is vital to neuronal activity. fMRI studies have demonstrated that the orbitofrontal cortex is involved in sensory integration[28], and hypoactivity in this area suggests that BSP patients are malfunctioning in sensorimotor integration, which may underlie the pathogenic mechanisms of BSP.

This finding is not completely consistent with previous findings obtained using fMRI or PET. Yang and colleagues have identified increased neural activity in the left orbitofrontal area (from the middle frontal gyrus to the inferior frontal gyrus) in a resting state fMRI study[16]. Similarly, Kerrison *et al*. conducted PET scans in patients with BSP and determined that the inferior frontal gyri (orbitofrontal area) exhibited increased glucose metabolism. However, decreased glucose uptake ventral to this region was also identified[15]. The inconsistency in these findings may have resulted from the different states in which our research was based, that is, the VFT compared with resting state fMRI and PET investigations.

### Psychiatric symptoms exhibit a limited impact on the prefrontal cortex

Regardless of the presence or absence of depression and anxiety symptoms in BSP, none of the channels were significantly different between the G1 and G2 groups. This finding indicates that psychiatric symptoms exhibit minimal, if any, impact on the prefrontal cortex in BSP patients. However, the reliability of this argument requires additional evidence from large-sample, double-blind, controlled studies.

### Neurobiological basis for BSP and mental disorders

The VFT has been used to determine prefrontal activation patterns in psychiatric patients compared with HC[26,29]. As a result of the hypoactivation in the lower frontal cortex[30] during the VFT in individuals with depressive disorders, there is a strong rationale to speculate that BSP and mental disorders may share a common neurobiological basis in the prefrontal cortex. Several studies have suggested that the basal ganglia-thalamo-cortical circuits may underlie both motor and emotional symptoms[31], which may support the increased prevalence of psychiatric disorders in BSP patients. However, these findings do not imply that depression or anxiety are caused by BSP or vice versa; rather, these common pathogenic mechanisms facilitate the development of one disorder in the presence of the other disorder. Through this neural circuit, disrupted neural activity in motor loops may have an influence on limbic loops, including the orbitofrontal cortex, which mediate cognitive and emotional symptoms[17]. Negative affective input from the amygdala and orbitofrontal area could also link to the thalamus and caudate nucleus, which regulate the blink reflex[32].

### Limitations

Although a covariance analysis was used to avoid the impact of age differences between BSP patients and HC, age-matching studies are still required to confirm these results. Limited by the number of BSP patients, random errors may be slightly increased compared with large-scale investigations. In addition, we could not eliminate a selection bias because we only interviewed outpatients, which indicates that some findings may not be generalized to BSP patients as a whole.

## Conclusion

In conclusion, our random, case-control, task-related NIRS study in BSP patients provides novel evidence that BSP may represent an independent attributional factor of prefrontal cortex dysfunction in the absence of psychiatric disorders. These findings may represent a novel pathophysiological mechanism for BSP and may provide insights regarding its treatment. Additional investigations are required to determine whether the basal ganglia-thalamo-cortical circuit could be treated as a neuropharmacological target for BSP.

## Supporting Information

S1 Dataset Raw data of demographic information, task performance, and NIRS. For the purpose of further understanding our study, detailed records of demography, task performance, and NIRS data were provided as supporting information. Because of the substantial data and calculation amounts, NIRS data were only provided as the oxy-Hb increase, which was calculated by the difference between the mean oxy-Hb of the task and pre-task period.
(XLS)
